# Individually designed ablation of low-voltage areas in persistent atrial fibrillation—a randomized controlled trial (IDEAL-AF): study design and rationale

**DOI:** 10.1093/ehjopen/oeaf037

**Published:** 2025-04-11

**Authors:** Astrid Paul Nordin, Mats Jensen-Urstad, Emmanouil Charitakis, Finn Åkerström, Henrik Almroth, Csaba Herczku, Jari Tapanainen, Niklas Höglund, Nikola Drca

**Affiliations:** Heart and Lung Disease Unit, Department of Medicine, Huddinge, Karolinska Institutet, Hälsovägen 13, S-141 86 Stockholm, Sweden; Department of Cardiology, Karolinska University Hospital, Hälsovägen 13, S-141 86 Stockholm, Sweden; Heart and Lung Disease Unit, Department of Medicine, Huddinge, Karolinska Institutet, Hälsovägen 13, S-141 86 Stockholm, Sweden; Department of Cardiology, Karolinska University Hospital, Hälsovägen 13, S-141 86 Stockholm, Sweden; Heart and Lung Disease Unit, Department of Medicine, Huddinge, Karolinska Institutet, Hälsovägen 13, S-141 86 Stockholm, Sweden; Department of Cardiology, Karolinska University Hospital, Hälsovägen 13, S-141 86 Stockholm, Sweden; Heart and Lung Disease Unit, Department of Medicine, Huddinge, Karolinska Institutet, Hälsovägen 13, S-141 86 Stockholm, Sweden; Department of Cardiology, Karolinska University Hospital, Hälsovägen 13, S-141 86 Stockholm, Sweden; Department of Cardiology, Linköping University Hospital, S-581 85 Linköping, Sweden; Department of Health, Medicine and Caring Sciences, Linköping University, S-581 85 Linköping, Sweden; Department of Cardiology, Sahlgrenska University Hospital, Blå Stråket 5, S-413 45 Gothenburg, Sweden; Department of Cardiology, Danderyd Hospital, Entrévägen 2, S-182 88 Stockholm, Sweden; Department of Cardiology, University Hospital of Umeå, Daniel Naezéns väg, S-907 37 Umeå, Sweden; Heart and Lung Disease Unit, Department of Medicine, Huddinge, Karolinska Institutet, Hälsovägen 13, S-141 86 Stockholm, Sweden; Department of Cardiology, Karolinska University Hospital, Hälsovägen 13, S-141 86 Stockholm, Sweden

**Keywords:** Low-voltage ablation, Persistent atrial fibrillation, Randomized study

## Abstract

**Aims:**

Voltage-based ablation is a promising catheter ablation strategy for atrial fibrillation (AF) in which low-voltage zones (LVZs) are targeted as a complement to pulmonary vein isolation (PVI). In a randomized setting, we intend to investigate whether PVI plus ablation of LVZs, compared to PVI-only, decreases the incidence of arrhythmia recurrence and improves health-related quality of life (HRQoL) in patients with persistent AF and LVZs.

**Methods and results:**

Individually designed ablation of low-voltage areas in persistent atrial fibrillation trial (IDEAL-AF; NCT04377594) is a multi-centre, randomized, controlled clinical trial. Patients with persistent AF and LVZs ≥ 3.0 cm² outside the PVI ablation lines will be randomized in a 1:1 ratio to either PVI or PVI plus LVZ ablation. The primary outcome will be the recurrence of atrial arrhythmias off anti-arrhythmic drugs during 12 months of follow-up after one to two ablation procedures within 6 months. A 3-month blanking period will be applied after the first procedure. Patients will be monitored using a smart phone-based ECG recording device throughout the follow-up period. With an anticipated enrolment of 936 patients, this study has 80% power to detect a 20% absolute risk reduction in the primary endpoint. Additionally, HRQoL improvement will be assessed using three questionnaires.

**Conclusion:**

IDEAL-AF is a multi-centre, randomized, controlled clinical trial investigating whether ablation of LVZs in addition to PVI reduces the recurrence rate of atrial arrhythmias and improves HRQoL compared to PVI-only in patients with persistent AF and LVZs. This study has the potential to modify recommendations regarding ablation techniques for this specific patient cohort.

## Introduction

Catheter ablation (CA) is an established treatment for patients with atrial fibrillation (AF). Eliminating pulmonary vein triggers through pulmonary vein isolation (PVI) is the cornerstone of ablation therapy. This approach is particularly successful in patients with paroxysmal AF and holds a Class I recommendation in both European and American guidelines.^1–3^ However, in many patients with AF, especially those with persistent AF, PVI-only often fails to achieve arrhythmia freedom. In these patients, the effectiveness of PVI-only has been notably low, with a success rate of ∼50% in an unselected population.^4–6^

Other ablation strategies, such as ablation of anatomically defined lines and ablation of complex fractionated atrial electrograms have been proposed to improve outcomes. However, randomized clinical trials (RCTs) have not demonstrated any additional benefit compared to PVI-only.^7,8^ In recent years, multiple studies have identified a correlation between the presence of atrial low-voltage zones (LVZs) and an increased rate of atrial arrhythmia recurrence after PVI.^9,10^ Fibrosis in the atrium disrupts cardiomyocyte connections, leading to slow conduction, which contributes to the initiation and maintenance of AF.^11^ Low amplitude of electrical potentials on electroanatomic mapping has been used as a surrogate marker for atrial fibrosis.^12^

A promising new strategy for AF ablation is thus voltage-based CA, which aims to modify low-voltage regions and convert them into electrically silent areas. Recent randomized studies investigating the ablation of LVZs in patients with persistent AF have shown conflicting results.^6,13,14^ In this randomized controlled study, we aim to determine whether PVI combined with LVZ ablation reduces the risk of arrhythmia recurrence and improves health-related quality of life (HRQoL) in patients with persistent AF and LVZs in the left atrium (LA) compared to PVI-only.

## Methods

### Study design and randomization process

**Figure 1:**
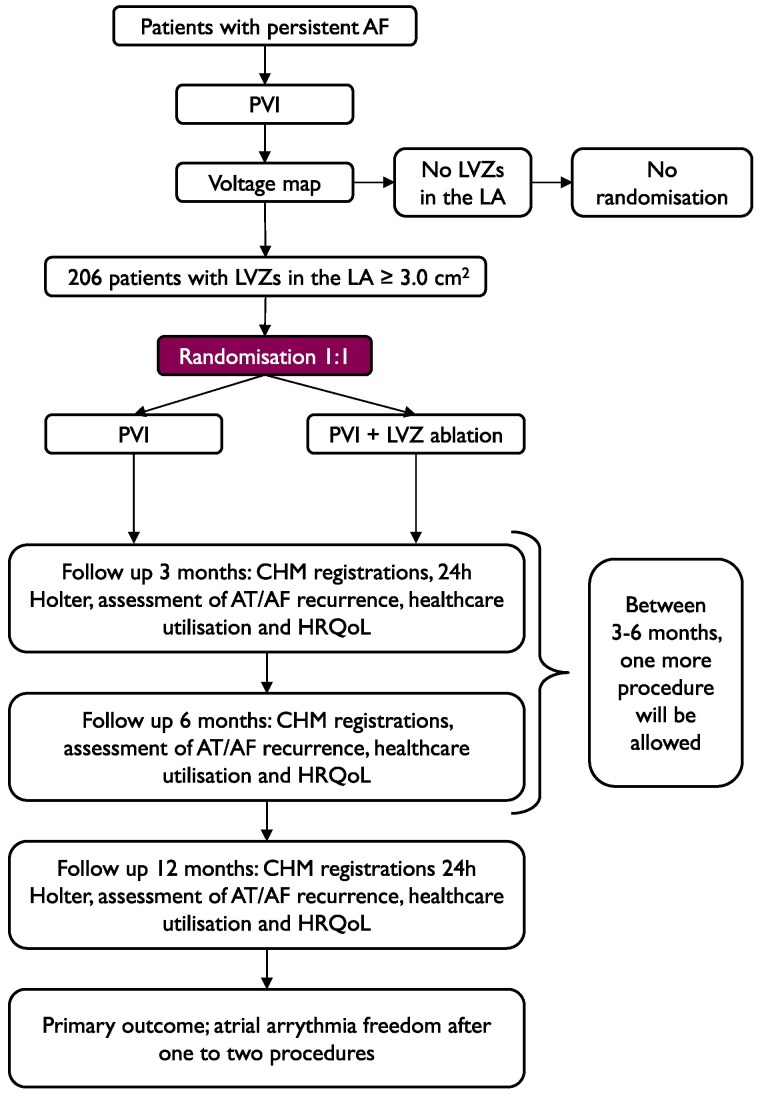
Flowchart for IDEAL-AF. AF, atrial fibrillation; AT, atrial tachycardia; CHM, Coala heart monitor; HRQoL, health realted quality of life; LA, left atrium; LVZ, low-voltage zone; PVI, pulmonary vein isolation.

The individually designed ablation of low-voltage areas in persistent atrial fibrillation trial (IDEAL-AF) (www.clinicaltrials.gov registration no. NCT04377594) is an investigator-initiated, prospective, randomized, multi-centre clinical trial. Five ablation centres in Sweden will participate: Karolinska University Hospital, Stockholm; Linköping University Hospital, Linköping; Sahlgrenska University Hospital, Gothenburg; Danderyd Hospital, Stockholm; and Umeå University Hospital, Umeå. The study flowchart is presented in *Figure 1*. Patients with symptomatic persistent AF who meet the clinical indications for AF ablation according to the EHRA consensus statement^15^ and are scheduled for first-time AF ablation will be included.

The inclusion and exclusion criteria are itemized in *Table 1*. The study will be single-blinded, meaning that patients will not be informed about the ablation strategy used, whereas the operator cannot be blinded due to the nature of the intervention. In patients presenting in sinus rhythm (SR), a local activation time (LAT) map will be collected simultaneously with the anatomical map. Patients presenting in AF will undergo electrical cardioversion to SR at the start of the procedure, followed by the collection of the LAT and anatomical maps. If the patient does not convert to SR, the ablation procedure will begin with PVI during AF, followed by another attempt at cardioversion after PVI. If the patient remains resistant to cardioversion, they will be excluded from the study, and any further ablation will be at the operator’s discretion.

**Table 1:** Study inclusion and exclusion criteria

**Inclusion criteria:**
ECG-documented persistent or long-standing persistent AF according to the European Society of Cardiology’s (ESC) definition. Symptoms, e.g. palpitations, dyspnoea, tiredness that is due to the atrial fibrillation. Suitable candidate for catheter ablation. Tried one or more anti-arrhythmic drugs or unwilling to try anti-arrhythmic drugs. Age ≥18 years.
**Exclusion criteria:** LA dimension >55 mm as determined by an echocardiography within the previous year. Acute coronary syndrome or coronary artery bypass surgery within 12 weeks. Severe aortic or mitral valvular heart disease using the ESC guidelines. Congenital heart disease. Prior surgical or percutaneous AF ablation procedure or atrioventricular-nodal ablation. Prior surgery involving LA. Medical condition likely to limit survival to < 1 year. NYHA class IV heart failure symptoms. Contraindication to oral anti-coagulation. Renal failure requiring dialysis. AF due to reversible cause. Pregnant and fertile women without anti-conception. History of non-compliance to medical therapy. Patients those are unable or unwilling to provide informed consent.

AF, atrial fibrillation; LA, left atrium; NYHA, New York Heart Association.

After PVI is completed (with confirmed entrance and exit block) and the patient is in SR, the high-density voltage map will be evaluated for LVZs—or collected and evaluated if the patient could not be cardioverted before PVI. This approach ensures that the operator remains blinded to the distribution of LVZs before PVI, preventing the placement of pulmonary vein (PV) lines from being influenced by LVZ locations.

**Figure 2:**
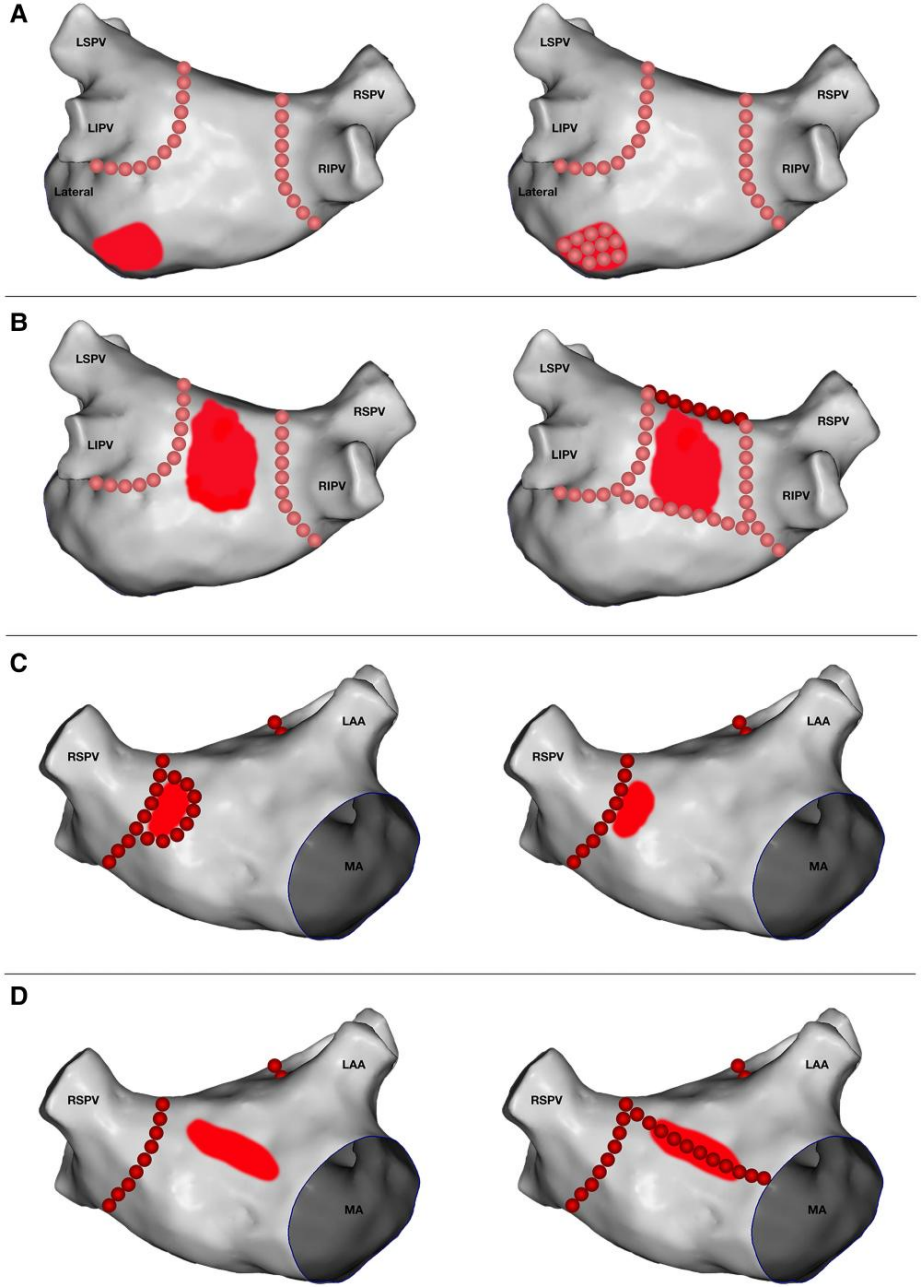
Strategies for low-voltage zones ablation. (*A*) Homogenization (PA view). (*B*) Box isolation (PA view). (*C*) Box isolation with connection to PV line (AP view). (*D*) Mitral line (AP view). AP, anteroposterior; LAA, left atrial appendage; LIPV, left inferior pulmonary vein; LSPV, left superior pulmonary vein; PA, posteroanterior; RIPV, right inferior pulmonary vein; RSPV, right superior pulmonary vein.

LVZs will be defined as areas with bipolar voltage <0.5 mV.^16,17^ Patients who exhibit at least one area of LVZ of ≥3.0 cm^2^ outside the encircling of the PVs will be classified as having a significant LA substrate. Patients with significant LA substrate (≥3.0 cm^2^) will be randomized (1:1) to (i) no additional ablation (PVI-only) or (ii) LVZ ablation (tissue homogenization, linear lesions, box isolation, or a combination of these) (*Figure 2*).^18^ Block randomization will be performed and stratified for sex (male and female) and LVZ size (3.0–14.9 cm^2^, 15.0–29.9 cm^2^, or ≥30 cm^2^). Randomization will be performed using Research Electronic Data Capture (REDCap)^19,20^ hosted at Karolinska Institutet. The Karolinska Institutet Biostatistics Core Facility has created the block randomization table, blinded to all participating centres and researchers.

### Ethical considerations

The Swedish ethical review authority has approved the study protocol and all data collection (no. 2019-05251, 2020-06319, 2022-02445-02, and 2023-02917-02). Patient data will be collected in accordance with institutional ethics guidelines and in compliance with the Declaration of Helsinki. All participants will receive both oral and written information about the study before providing their written informed consent. Study data will be pseudonymized and stored in REDCap, version 14.0.43, a secure electronic data capture tool hosted at Karolinska Institutet. The Department of Cardiology at Karolinska University Hospital will serve as the co-ordinating centre for this study. Safety endpoints, along with all other adverse events (AEs), will be continuously monitored, documented, and assessed for association with study treatment by the principal investigator and co-investigators.

All participating centres are required to report AEs to the co-ordinating centre and log them into REDCap. Dedicated research nurses will continuously conduct internal monitoring throughout the study. The co-ordinating centre will review voltage map samples from all participating centres. No interim analyses are planned.

### Amendments to protocol

No amendments have been made to the ablation or the follow-up protocol. However, the Swedish Ethical Review Authority has approved the following changes to the study protocol:

- Inclusion of urinary tests and Helicobacter pylori testing for study participants.
- Addition of Karolinska Institutet as a sponsoring institution alongside Karolinska University Hospital.
- Addition of Danderyd Hospital as an inclusion site.
- The possibility to perform computed tomography (CT) and magnetic resonance imaging (MRI) of the heart.

### Pre-procedural and intra-procedural assessment

During the pre-enrolment process, all patients will provide written informed consent, and detailed information regarding their medical history will be collected. Additionally, HRQoL questionnaires will be administered. Prior to the ablation, transthoracic echocardiography and oral anti-coagulation for a minimum of three weeks is mandatory. Transoesophageal echocardiography or CT will be performed to rule out the presence of thrombi in the left atrial appendage (LAA). Standard biochemical tests will be conducted, along with N-terminal pro–B-type natriuretic peptide (NT-proBNP) and high-sensitivity C-reactive protein (hs-CRP). Prior to the ablation procedure, three HRQoL questionnaires will be administered: the atrial fibrillation effect on quality-of-life (AFEQT) (AF-specific QoL), the RAND 36-Item Health Survey (RAND 36) (general QoL), and the Arrhythmia-Specific questionnaire in Tachycardia and Arrhythmia (ASTA) (arrhythmia-specific-QoL).

The procedure will be carried out in accordance with international and local standards, with heparin administered to maintain an activated clotting time at over 300 s. Prior to ablation, pressures in the right and left atria will be measured using a long sheath positioned in each atrium. In a subgroup of patients, blood samples will be collected from a short introducer in the femoral vein and from the left atrium, which will then be stored in a biobank.

### Assessment of low-voltage zones in left atrium

An endocardial bipolar voltage map of the LA will be acquired using multi-polar mapping catheters (PENTARAY or OCTARAY, CARTO, Biosense Webster Inc., CA, USA) during pacing in the coronary sinus (CS) at 600 milliseconds (ms). The mapping settings will be as follows: LAT stability of 4–5 ms, cycle length stability of 590–610 ms, and tissue proximity index activated. Areas with bipolar voltage <0.5 mV will be defined as LVZs, and an interpolation threshold with a maximum distance of 5 mm will be used for surface colour projection. Thorough coverage of all areas of the LA will be required. Since CS-pacing can create false-positive LVZs near the pacing pole, such areas will be confirmed by creating a voltage map during SR. LVZ up to 3.0 cm^2^ adjacent to the transseptal puncture will be subtracted from this area to avoid incorrectly defining the foramen ovale as diseased tissue.^21^ LVZs ≥ 3.0 cm^2^ will be confirmed using an ablation catheter (THERMOCOOL SMARTTOUCH, THERMOCOOL SMARTTOUCH SF, or QDOT MICRO, Biosense Webster Inc, CA, USA) with a contact force of 5–30 g.

### Intervention

#### Pulmonary vein isolation

The operators, blinded to the voltage map, will perform antral PVI using a contact force-sensing irrigated tip catheter with a maximum power of 50W (THERMOCOOL SMARTTOUCH, THERMOCOOL SMARTTOUCH SF or QDOT MICRO, Biosense Webster Inc., CA, USA).^22,23^ In short, the procedure involves placing the ablation lesions point by point continuously in a circle around the pulmonary vein pairs, according to the CLOSE-protocol.^22^

Visualization of RF applications (Visitag®, Biosense Webster Inc., CA, USA) will be used to mark the ablation points, targeting an inter-lesion distance < 6 mm. Entrance and exit block in all pulmonary veins will be confirmed using standard techniques at the end of the procedure, employing a multi-polar mapping catheter (PENTARAY or OCTARAY) inside the PVs.^22,23^

#### LVZ ablation

LVZ ablation is recommended to be carried out in one of three ways, depending on the area’s position and at the operator’s discretion, as illustrated in *Figure 2*:

- **Homogenization**: This involves regional ablation of the LVZs. The endpoint is achieved through a significant reduction of local electrograms, defragmentation, and loss of ability to excite the underlying ablated tissue when stimulated with 10 V@2 ms or more.
- **Box isolation**:
  - Example 1: LVZs in the posterior wall; a roof line and a posterior line between the lower pulmonary veins can isolate the entire posterior wall.
  - Example 2: LVZs in the anterior wall; a roof line is ablated connecting the left superior pulmonary vein (LSPV) and right superior pulmonary vein (RSPV), and one septal and one anterior mitral line is ablated connecting the mitral valve and RSPV and LSPV.
  - Example 3: LVZs near a PV encircling; the circle can be expanded to incorporate the LVZ.
  - In this method, entrance and exit block must be confirmed by pacing within the box at 10V@2 ms or more.
- **Lines:** This includes the roof line, anterior mitral line, septal line, and lateral mitral line. If LVZs are identified in regions where a critical isthmus could be created, lines should be ablated to avoid future macroreentrant atrial arrhythmias. Bidirectional block over the lines must be confirmed using standard pacing manoeuvres.
- **Combination:** The above methods can be combined as needed.

#### Post-ablation induction

After completion of PVI or PVI + LVZ ablation (depending on the randomization), a 10-s period burst pacing protocol is initiated, pacing from the proximal CS, starting at 300 ms, with decrements of 20 ms down to 200 ms or until atrial refractoriness is reached.

In patients randomized to PVI + LVZ ablation, any induced regular arrhythmias such as atrial flutter (AFL) or atrial tachycardia (AT) will be mapped and ablated. If AF is induced, the patient will be electrically converted to SR. In patients randomized to PVI-only, electrical cardioversion will be performed for all atrial arrhythmias except for typical cavotricuspid isthmus-dependent AFL, which will be ablated.

If the patient presents with atypical AFL at the start of the procedure, it must be ablated in patients randomized to PVI + LVZ ablation. In patients randomized to PVI-only, mapping and ablation of the flutter is optional but not required.

### Follow-up and patient management

Follow-up visits will be scheduled 3, 6, 12, 18, and 24 months after the first ablation procedure. Only the randomized patients (*n* = 206) and the 103 non-randomized control patients will be followed up in the study. HRQoL questionnaires, including RAND-36, AFEQT, and ASTA, will be administered at all follow-up appointments. All patients will be provided with a Coala Heart Monitor (CHM, Coala Life AB, Uppsala, Sweden) for 2 years to monitor their heart rhythm. CHM is a hand-held device that records a 30-s chest- and a 30-s thumb ECG.^24^ Participants will be instructed to record ECG (chest + thumb) whenever they experience symptoms indicative of atrial arrhythmia recurrence, as well as twice daily for 14 days before each follow-up visit. Additionally, 24-h ambulatory Holter monitoring will be performed at 3 and 12 months. If the patient has a pacemaker (PM) or an implantable cardiac defibrillator (ICD) with atrial lead and cannot handle the CHM device, follow-up will be monitored by the PM or ICD. Arrhythmia recurrence will be defined as documented AF or AT lasting >30 s after a blanking period of 90 days post-ablation. Documentation is mandatory through Coala Heart Monitor, e-patch, 12-lead ECG, Holter or PM/ICD, or other ambulatory rhythm control devices.

Anti-arrhythmic medications (except beta-blockers and calcium channel blockers) will be discontinued 4–6 weeks after the ablation procedure, with the exception of amiodarone, which will be discontinued immediately after the procedure. All patients will be followed up at their respective ablation centres. In the case of arrhythmia recurrence, anti-arrhythmic medications may be reinitiated based on clinical judgment. If arrhythmia recurs after the 90-day blanking period but within 6 months of the index procedure, reablation will preferably be scheduled within 6 months from the index procedure. Follow-up for the primary outcome in patients undergoing reablation will be conducted 12 months after the first ablation procedure.

Personnel blinded to treatment allocation will analyse and classify all CHM registrations, Holter registrations and all other ECGs according to arrhythmia outcomes (AF, AT, or SR).

NT-proBNP and hs-CRP levels will be analysed at the 12-month follow-up.

### Outcomes

#### Primary outcome

Freedom from documented atrial arrhythmia off anti-arrhythmic medications (except beta-blockers or calcium channel blockers), including AF or AT, lasting > 30 s after one to two ablation procedures within 6 months will be assessed at 12 months follow-up. A blanking period of 90 days after the first procedure, but not after a potential second procedure, will be applied. Each occurrence of atrial arrhythmia in a patient, regardless of the allocation group, will be counted as a treatment failure.

#### Safety outcomes and adverse events

Safety outcomes include cardiac perforation, tamponade, stroke, transient ischaemic attack, systemic embolic events, atrial-oesophageal fistula, major bleeding, atrioventricular block, PV stenosis, groin hematoma requiring vascular intervention, or extended hospitalization within 90 days following the ablation procedure. All-cause mortality will be assessed at 12 and 24 months after the first ablation procedure.

#### Secondary outcomes

Pre-specified main secondary outcomes:

- Freedom from documented atrial arrhythmias >30 s at 12 months after one ablation procedure without anti-arrhythmic drugs. Blanking period: 90 days after the first procedure.
- Freedom from documented atrial arrhythmias >30 s at 12 months after one ablation procedure with or without anti-arrhythmic drugs. Blanking period: 90 days after the first procedure.
- Freedom from documented atrial arrhythmias >30 s at 12 months after one or two ablation procedures within 6 months with or without anti-arrhythmic drugs. Blanking period: 90 days after the first procedure, but no blanking period after a potential second procedure.
- Changes between groups in HRQoL assessed with AFEQT, ASTA, and RAND-36 at 3, 6, 12, 18, and 24 months compared with HRQoL before the index procedure.
- Differences between groups concerning procedural and fluoroscopy time.

All pre-specified outcomes of the study are detailed in Supplementary material online, *Table S1*.

#### Pre-specified sub-studies

Pre-specified sub-studies are presented in Supplementary material online, *Table S2*.

### Statistics

#### Sample size estimation

We conducted a sample size calculation for the primary outcome: freedom from atrial arrhythmia off anti-arrhythmic drugs at 12-month follow-up in the LVZ + PVI ablation group compared to the PVI-only group. This calculation was based on two previous studies by Rolf *et al*. and Kircher *et al*.^18,25^ Based on these studies, the estimated absolute risk reduction in arrhythmia freedom at 12 months between the two groups was projected to be 20%. Accounting for an estimated 10% loss to follow-up, an assumed power of 80%, a significance level of 0.05, and the assumption that 22% of patients with persistent AF have an LVZ >3 cm², we determined that 936 patients with persistent AF would need to be screened and ablated to randomize 206 patients (103 per group) to detect a statistically significant difference between the two groups.

#### Statistical analyses

The primary analysis will be based on the intention-to-treat principle, while a per-protocol analysis will be conducted as part of the sensitivity analyses. The primary outcome will be assessed using unadjusted Cox regression analysis. Descriptive statistics (clinical and demographic characteristics) will be provided by randomized treatment assignment. Furthermore, normally distributed data will be presented as means ± standard deviations for continuous variables and as counts and percentages for categorical variables with 95% confidence intervals, while non-normally distributed variables will be presented as medians and inter-quartile ranges. Statistically significant differences in baseline characteristics between the two randomization groups and the control group without significant LVZs will be evaluated using Pearson’s χ^2^ test or Fisher’s exact test for categorical data, Students’ *t*-test for normally distributed data, and the Wilcoxon rank sum test for non-parametric data. All *P*-values will be two-sided, and a *P*-value of < 0.05 will be considered statistically significant. All analyses will be performed using STATA/SE version 16.1 (Stata Corporation).

### Study management funding and timeline

#### Timeline

Patient inclusion began on 19 May 2020, and as of 17 February 2025, 878 patients have been ablated within the study, with 197 randomized.

## Discussion

The optimal strategy for ablating persistent AF in different patient categories has yet to be established. Despite a limited success rate, PVI remains the first-line ablation strategy for patients with persistent AF and currently holds a Class I recommendation in the International expert consensus statement on CA.^3^ Atrial fibrosis can generate and perpetuate AF through various arrhythmogenic mechanisms, both by acting as a trigger and maintaining re-entry circuits.^11,26^ Although LVZs are frequently regarded as surrogate markers for fibrosis, extensive research conducted over several years has consistently demonstrated that the presence of LVZs independently predicts the recurrence of AF following PVI.^9,25,27^ The specific extent of LVZs that increase the risk of arrhythmia recurrence following PVI remains vaguely defined. However, the risk of arrhythmia recurrence appears to rise with the enlargement of LVZs.^28,29^ In our study, we have implemented a meticulous process for selecting LVZs, explicitly targeting contiguous areas of ≥3 cm^2^. This decision was made to exclude patients with smaller LVZs who might not necessarily be at an increased risk of arrhythmia recurrence, thereby ensuring the accuracy of our findings.

CA of LVZs has been the subject of several studies with conflicting results, and numerous non-randomized studies suggest a benefit of LVZ ablation.^25,30,31^ However, fewer studies have been published in the RCT setting. The ERASE AF study evaluated PVI-only vs. PVI combined with LVZ ablation in persistent AF (*n* = 324). At the 12-month follow-up, the LVZ + PVI ablation group demonstrated significantly better arrhythmia-free survival.^6^ In contrast, Yang *et al*. conducted a similar study and found no significant difference in arrhythmia freedom between PVI-only and PVI + LVZ ablation (*n* = 300) at 18 months; however, this study was underpowered due to low LVZ-burden.^13^ The SUPPRESS AF^14^ (yet to be published) similarly found no significant difference between the PVI-only and PVI + LVZ-ablation groups. However, a sub-analysis in this study showed that patients with an enlarged left atrium had significantly better outcomes in the PVI + LVZ-ablation group. Finally, the DECAAF II trial^32^ targeted fibrosis guided by MRI. Patients were randomized to PVI-only or to PVI + MRI-guided fibrosis ablation (*n* = 843). This trial found no significant difference in arrhythmia freedom, with more severe complications occurring in the fibrosis-ablated group. Although MRI may detect atrial fibrosis, it is a more complex method compared to endocardial mapping and may not offer directly comparable results.^33^ The most widely available and practical mapping method is endocardial mapping using multi-polar catheters,^34^ which we will employ in our study.

In the ERASE-AF,^6^ STABLE-SR II^13^, and DECAAF,^35^ patients were randomized prior to the ablation procedure. As a result, a substantial proportion of these patients did not have LVZs in the LA and only underwent PVI. In our study, randomization will occur after the voltage map has been collected and after the completion of PVI. Since the operators will be blinded to the distribution of the LVZs, the layout of the PVI lines is not influenced by them. This approach guarantees that the design of the PV lines remains unaffected by the LVZ distribution. Additionally, it ensures that only patients with LVZs measuring ≥3.0 cm² are included in the randomization process, facilitating an equal distribution of patients across the randomization arms. Moreover, by stratifying participants according to sex and the size of the LVZs, we aim to achieve balanced proportions of women and men and a uniform representation of small, medium, and large LVZ sizes in both study arms. These measures are intended to enhance the reliability of our results. Furthermore, we have decided to allow one redo procedure (within 6 months) when evaluating the primary outcome of our study. This decision acknowledges that both PV lines and other ablated areas may experience reconnections. Since reablation is common in clinical practice, our approach thus reflects current clinical standards.

## Summary

The IDEAL-AF study is a multi-centre, single-blinded, randomized clinical trial. The primary aim is to assess the efficacy and safety of LVZ ablation in addition to PVI, compared to PVI-only, in achieving arrhythmia freedom at 12 months in patients with persistent AF.

## Supplementary Material

oeaf037_Supplementary_Data

## Data Availability

No new data were generated or analysed in support of this research.

## Lead author Biography

Dr. Astrid Paul Nordin, M.D., Ph.D., obtained her medical degree from Linköping University, Sweden, and completed her residency in internal medicine and cardiology at Södersjukhuset, Stockholm. She is currently an Associate Senior Consultant in the Department of Electrophysiology at Karolinska University Hospital, Stockholm, Sweden. In 2024, she defended her doctoral thesis titled ‘Catheter Ablation of Atrial Fibrillation—Methods for Improving Outcome.’
