# Supplementary material for: Individually designed ablation of low-voltage areas in persistent atrial fibrillation—a randomized controlled trial (IDEAL-AF): study design and rationale
Source: Eur Heart J Open. 2025 Apr 11;5(2):oeaf037. doi: 10.1093/ehjopen/oeaf037 (PMC12038156; doi:10.1093/ehjopen/oeaf037)
Supplement: oeaf037_Supplementary_Data [file oeaf037_supplementary_data.zip › Supplement - clean copy.docx]

Table S1. Secondary outcomes

- Freedom from documented atrial arrhythmias >30 seconds at 12 months after one ablation procedure without antiarrhythmic drugs. Blanking period: 90 days after the first procedure.
- Freedom from documented atrial arrhythmias >30 seconds at 12 months after one ablation procedure with or without antiarrhythmic drugs. Blanking period: 90 days after the first procedure.
- Freedom from documented atrial arrhythmias >30 seconds at 12 months after one or two ablation procedures within six months with or without antiarrhythmic drugs. Blanking period: 90 days after the first procedure but no blanking period after a potential second procedure.
- Freedom from documented AF >30 seconds at 12 months after one or two ablation procedures without antiarrhythmic drugs. Blanking period: 90 days after the first procedure but no blanking period after a potential second procedure.
- Freedom from documented symptomatic atrial arrhythmias >30 seconds at 12 months after one or two ablation procedures with or without antiarrhythmic drugs. Blanking period: 90 days after the first procedure but no blanking period after a potential second procedure.
- Freedom from documented atrial arrhythmias >30 seconds at 24 months after one to multiple ablation procedures without antiarrhythmic drugs.
- Freedom from documented symptomatic atrial arrhythmias >30 seconds at 24 months after one or multiple ablation procedures with or without antiarrhythmic drugs.
- Time from first ablation procedure to first documented recurrence of atrial arrhythmia.
- Achievement of no repeat procedures required 12 months following the index procedure.
- Changes between groups in HRQoL assessed with AFEQT, ASTA and RAND-36 at 3, 6, 12, 18 and 24 months compared with HRQoL before the index procedure.
- Differences between groups concerning the procedural and fluoroscopy time.
- Assessment of differences in the presence and distribution of LVZs between patients receiving and not receiving antiarrhythmic drugs.

Table S2. Prespecified sub-studies

- Follow-up of arrhythmia freedom and HRQoL at 24 months for all randomised patients
- Follow-up of non-randomised patients to determine whether those with transition zones (voltage <1.0 mV) experience a higher rate of arrhythmia recurrence compared to those without transition zones.
- Comprehensive follow-up assessment of changes in HRQoL in the IDEAL-AF cohort.
- The ability of echocardiography, CT data, and/or biomarkers to predict procedural success, and the presence of LVZs will be assessed.
- The potential of pre-procedural ECG measurements to predict the existence of LVZs, and the procedural outcome will be evaluated.
- Evaluation of whether a dilated sinus of Valsalva or ascending aorta can predict the presence of LVZs in the anterior wall.
- The ability of artificial intelligence (AI) processing of CARTO mapping images, and preprocedural data to predict procedural success will be assessed.
